# Differential associations between mentalizing dimensions and psychopathy subtypes: the moderating role of borderline personality traits

**DOI:** 10.3389/fpsyg.2025.1685417

**Published:** 2025-10-15

**Authors:** Buket Ünver

**Affiliations:** Department of Psychology, Faculty of Economics, Administrative and Social Sciences, Işık University, Istanbul, Türkiye

**Keywords:** psychopathy, mentalizing, borderline personality, motivation to mentalize, personality disorders

## Abstract

**Introduction:**

Psychopathy comprises primary and secondary subtypes with distinct affective–interpersonal profiles. Mentalizing, i.e., the capacity to understand one’s own and others’ mental states, may help explain this heterogeneity. This study tested how three mentalizing dimensions (Self-Related, Other-Related, and Motivation to Mentalize) relate to psychopathy subtypes and whether borderline personality traits (BPTs) moderate these associations.

**Methods:**

Adults from a community sample (*N* = 953) completed validated measures of psychopathy, mentalizing, and BPTs. BPTs were modeled as a continuous variable. Multivariable linear regressions predicted primary and secondary psychopathy from the three mentalizing facets while adjusting for age, gender, socioeconomic status, and psychiatric diagnosis. Moderation was examined via interaction terms between each mentalizing facet and BPTs; significant interactions were probed at −1/0/+1 SD of BPT scores.

**Results:**

Higher Motivation to Mentalize and greater Self-Related Mentalizing were uniquely associated with lower primary psychopathy; Other-Related Mentalizing was not a unique predictor. For secondary psychopathy, Self-Related Mentalizing and, to a lesser extent, Motivation to Mentalize were inversely associated; Other-Related Mentalizing was not significant. BPTs significantly moderated only the association between Motivation to Mentalize and primary psychopathy (stronger inverse association at higher BPTs); no moderation effects emerged for secondary psychopathy.

**Conclusion:**

Findings indicate that motivation and self-related aspects of mentalizing are protective correlates of psychopathic traits, with moderation by BPTs limited to primary psychopathy. Targeting motivation to consider mental states and strengthening self-reflective capacity may enhance psychological intervention strategies, particularly for individuals high in primary psychopathy with elevated borderline features.

## Introduction

Psychopathy is a multidimensional personality construct characterized by deficits in empathy, emotional coldness, impulsivity, deviation from moral norms, and patterns of antisocial behavior (De Brito et al., 2021; Hare, 2003; Salekin et al., 2010). Although traditionally associated with criminal behavior, psychopathic traits have also been observed in both community and clinical populations (Sanz-García et al., 2021). Contemporary theoretical approaches conceptualize psychopathy not as a homogeneous entity but as a spectrum involving distinct cognitive and emotional mechanisms, generally classified into two subtypes: primary and secondary psychopathy (Berg et al., 2013; Hicks and Drislane, 2018; Patrick, 2022; Skeem et al., 2007).

Primary psychopathy is characterized by low fear reactivity, affective detachment, lack of empathy, and instrumental aggression (Hofmann et al., 2021; Lykken, 1995; Skeem et al., 2007). Individuals with this profile often exhibit insensitivity to others’ emotions—particularly fear—and show impairments in recognizing facial expressions of affect (Blair et al., 2004; Dadds et al., 2006; De Brito et al., 2021; Kyranides et al., 2022). In contrast, secondary psychopathy is defined by high emotional reactivity, inner distress, and impulsivity, along with disorganized or exaggerated empathic responses (Campos et al., 2023; Decety et al., 2013; Hare, 2003; Hofmann et al., 2021). This profile is also marked by difficulties in emotion regulation, inconsistent moral reasoning, and impulsive aggression (Gregory et al., 2012; Sellbom and Drislane, 2021). Furthermore, similar to primary psychopathy, individuals with secondary psychopathic traits also exhibit impairments in recognizing emotions in facial expressions and evaluating others’ mental states (Dolan and Fullam, 2004; Vonk et al., 2015). Particularly, deficits in accurately inferring the intentions, beliefs, and emotional experiences of others may lead to significant disruptions in social functioning. A comparative overview of the distinguishing features of primary and secondary psychopathy, together with their related mentalizing deficits, is presented in Table 1. Lastly, growing evidence indicates that psychopathy is also linked to diminished mentalizing capacity (Carroll et al., 2021; Song et al., 2023). However, knowledge regarding how this link operates—especially in relation to secondary psychopathy—remains limited.

**Table 1:** Characteristics of primary vs. secondary psychopathy and related mentalizing deficits.

Dimension	Primary psychopathy	Secondary psychopathy
Core features	Low fear reactivity, affective detachment, lack of empathy, instrumental aggression (Hofmann et al., 2021; Lykken, 1995; Skeem et al., 2007)	High emotional reactivity, inner distress, impulsivity, disorganized or exaggerated empathic responses (Hare, 2003; Hofmann et al., 2021; Campos et al., 2023; Decety et al., 2013)
Emotional & interpersonal traits	Insensitivity to others’ emotions—particularly fear; deficits in recognizing facial expressions (Blair et al., 2004; Dadds et al., 2006; De Brito et al., 2021; Kyranides et al., 2022)	Difficulties in emotion regulation, inconsistent moral reasoning, impulsive aggression (Gregory et al., 2012; Sellbom and Drislane, 2021)
Mentalizing deficits	Negative link with Other-Related Mentalizing; diminished responsiveness to affective cues (Bo et al., 2023; Song et al., 2023); reduced motivation to engage with emotional content; cognitive perspective-taking often used manipulatively (Baskin-Sommers and Brazil, 2022; De Wit-De Visser et al., 2023; Lukacs et al., 2022; Tillem et al., 2021)	Deficits in Self-Related Mentalizing linked to emotion regulation difficulties (Sheikhi and Aminiha, 2022; Hare, 2003); emotional awareness deficits; high negative affectivity, hostility, and impulsivity linked to difficulties in understanding both self and others’ emotions (Smith et al., 2022; Gómez-Leal et al., 2018)

Mentalizing refers to the capacity to interpret one’s own and others’ emotions, thoughts, beliefs, and intentions (Fonagy and Bateman, 2019; Lüdemann et al., 2021). Diminished mentalizing capacity has been associated with behavioral problems such as aggression and impulsivity, whereas increased mentalizing may buffer against these tendencies (Velotti et al., 2021; Yakeley and Williams, 2014), also in individuals with psychopathic tendencies (Taubner et al., 2013). Mentalizing capacity is often impaired in individuals with personality disorders and has been linked to difficulties in emotional regulation and inconsistencies in self-perception (Bateman and Fonagy, 2016; Newbury-Helps et al., 2017). These impairments are especially prominent in individuals with comorbid psychopathy and antisocial personality disorder (Dolan and Fullam, 2004; Newbury-Helps et al., 2017).

When examined across its dimensions, mentalizing comprises three primary components: Self-Related Mentalizing, Other-Related Mentalizing, and Motivation to Mentalize (Törenli-Kaya et al., 2023). Self-Related Mentalizing refers to the ability to understand one’s own desires, needs, emotions, and intentions (Arabadzhiev and Paunova, 2024; Fonagy and Allison, 2013). Other-Related mentalizing involves the capacity to interpret the mental states of others (Fonagy and Allison, 2013; Fonagy and Luyten, 2009; Fonagy et al., 2018). Motivation to Mentalize refers to the individual’s intrinsic drive to engage in mentalizing processes (Dimitrijević et al., 2018; Stefana et al., 2024).

Recent research has begun to clarify how these subcomponents relate to psychopathy. For example, primary psychopathy has been associated with deficits in theory of mind skills and difficulty recognizing others’ perspectives and feelings (Gillespie et al., 2017; Song et al., 2023). This suggests a potential negative link between primary psychopathy and Other-Related Mentalizing (Bo et al., 2023). Interestingly, despite their affective detachment, individuals with primary psychopathic traits may still interpret others’ intentions and goals for manipulative purposes (Lukacs et al., 2022). In contrast, deficits in Self-Related Mentalizing have been linked to emotion regulation difficulties and are conceptually aligned with secondary psychopathy (Hare, 2003; Sheikhi and Aminiha, 2022). Emotional awareness has been found to be negatively associated with secondary psychopathy, and to a lesser extent, with primary psychopathy (Gómez-Leal et al., 2018; Smith et al., 2022). These findings suggest that individuals with high levels of negative affectivity, hostility, and impulsivity may have difficulties in understanding both their own internal states and the emotions of others. Lastly, Motivation to Mentalize has also emerged as a relevant factor in psychopathy. Individuals with psychopathic traits often show reduced interest in understanding others’ mental states, and those with primary psychopathic features appear to struggle more with emotional content than cognitive content (Baskin-Sommers and Brazil, 2022; De Wit-De Visser et al., 2023; Lukacs et al., 2022). This may reflect a tendency to focus selectively on information that serves personal goals while ignoring affective cues (Tillem et al., 2021). In cases where psychopathy co-occurs with schizotypal traits, Motivation to Mentalize may still be intact, but often directed toward manipulative or exploitative ends (Baskin-Sommers and Brazil, 2022; Gillespie et al., 2021).

These processes become even more complex in the context of borderline personality patterns, which overlap significantly with psychopathy in terms of low mentalizing capacity, impulsivity, and affective instability (Fonagy and Luyten, 2009; Howard et al., 2014). Studies have shown that both primary and secondary psychopathy are associated with borderline traits (López-Villatoro et al., 2020; Stålenheim and Knorring, 1998). While primary psychopathy aligns with deficits in empathy and egocentrism, secondary psychopathy shares features such as emotional dysregulation, impulsivity, and unstable interpersonal functioning with borderline personality disorder (BPD) (Babcock and Michonski, 2019; Burns et al., 2015; Penson et al., 2016; Vaillancourt and Brittain, 2019). Some researchers have even proposed that psychopathy may represent a phenotypic expression of BPD (Carlisle, 2014; Sprague et al., 2012), with low boldness and high levels of meanness and impulsivity being associated with more severe borderline symptomatology (Chapman, 2019; Daurio and Taylor, 2022).

These findings indicates notable gaps in the current literature, particularly regarding how specific dimensions of mentalizing relate to different subtypes of psychopathy, and how these relationships may vary in the presence of borderline personality features. Addressing this gap requires more differentiated models of clinical assessment and intervention tailored to individual personality configurations. Exploring how mentalizing capacity intersects with the distinct cognitive and emotional profiles of psychopathy subtypes may enhance understanding of the mechanisms underlying psychopathic tendencies and inform targeted, effective interventions.

This study examined relationships among three dimensions of mentalizing (i.e., Self-Related, Other-Related, and Motivation to Mentalize) and two psychopathy subtypes (primary versus secondary) in the full sample. Borderline personality traits were treated as a continuous variable. We estimated multivariable linear models including all mentalizing facets and covariates, and we tested the moderating role of borderline traits using interaction terms between each mentalizing facet and borderline trait severity. Simple slopes were probed for all interactions (whether significant or not) at −1, 0, and +1 SD of borderline traits. This approach clarifies how each dimension of mentalizing relates to psychopathy subtypes across the continuum of borderline features, informing targeted interventions that distinguish difficulties in emotional self-regulation from cognitive-empathic processes.

## Methods

### Participants and procedure

The sample consisted of 953 volunteer adults. Participants ranged in age from 18 to 68 years (*M* = 33.6, SD = 12.4), with women comprising 43% of the sample. Among them, 154 reported having at least one psychiatric diagnosis, and 92 reported taking psychotropic medications. The inclusion criterion for the study was a minimum age of 18 years; no exclusion criteria were utilized. Data were collected using the snowball sampling method, and the entire data collection process was conducted via Google Forms. The Google Form also included an informed consent form that participants were required to accept before proceeding with the survey. The survey was primarily disseminated via social media platforms (e.g., WhatsApp, Instagram), with initial recruitment facilitated through undergraduate and graduate students, who received research participation credit for their involvement and for inviting further participants. The survey questionnaire was administered in the Turkish language. Table 2 presents the sociodemographic and clinical information.

**Table 2:** Demographic and clinical characteristics of participants.

Variable	Level	*N* = 953
Age, years (M, SD)		31.75 (2.2)
Gender	Women	407 (43%)
	Men	545 (57%)
Education	Non-binary	1 (0%)
	Lower-secondary	110 (12%)
	High-school	717 (75%)
	University Degree	126 (13%)
Socioeconomic status	Level 1	15 (2%)
	Level 2	103 (11%)
	Level 3	575 (60%)
	Level 4	236 (25%)
	Level 5	24 (3%)
Psychiatric diagnosis	None	799 (84%)
	Any	154 (16%)
Psychiatric medication	No	861 (90%)
	Yes	92 (10%)

Socioeconomic status was assessed through self-reported perceived socioeconomic level on a 5-point scale (1 = lowest, 5 = highest).

### Measures

#### Sociodemographic and clinical characteristics

The form was used to obtain information regarding participants’ age, gender, educational level, socioeconomic status, and clinical characteristics (e.g., psychiatric diagnoses and psychotropic medication use).

#### Borderline personality questionnaire (BPQ)

The Borderline Personality Questionnaire (BPQ; Poreh et al., 2006) is used to evaluate borderline personality traits. The Turkish adaptation of the scale was conducted by Ceylan et al. (2017). The scale comprises 80 items. The scale has 9 subscales: impulsivity, instability in affect, abandonment, relationships, self-image, suicide/self-mutilation behavior, emptiness, intense anger and psychosis like case. Scores that are higher indicate the presence of borderline features to a greater extent (Ceylan et al., 2017). In the current sample, McDonald’s ωₜ demonstrated excellent reliability for the total scale (0.98) and ranged from acceptable to excellent (0.79–0.91) across the nine subscales.

#### Mentalization scale (MentS)

The Mentalization Scale (MentS; Dimitrijević et al., 2018) is used to assess mentalizing capacity in both clinical and community samples. The Turkish adaptation of the scale was conducted by Törenli-Kaya et al. (2023). The scale consists of 25 items and includes three subscales. In addition to providing a total score, it allows for the assessment of the following subscales: Self-Related, Other-Related, and Motivation to Mentalize (Törenli-Kaya et al., 2023). In the present sample, McDonald’s ωₜ demonstrated high reliability for the full scale (0.91) as well as for the Self-Related (0.83) and Other-Related (0.88) subscales, and acceptable reliability for the Motivation subscale (0.77).

#### Levenson self-report psychopathy scale (LSRP)

Levenson Self-Report Psychopathy Scale (LSRP; Levenson et al., 1995) is used to assess psychopathy. The Turkish adaptation of the scale was conducted by Engeler and Yargıç (2004). The scale consists of 26 items. It has two subscales: primary psychopathy and secondary psychopathy. The subscale scores are calculated separately and can be added together to give a total score (Engeler and Yargıç, 2004). In our sample, internal consistency was high for the total scale (McDonald’s ωₜ = 0.88) and primary subscale (ωₜ = 0.90), and acceptable for the secondary subscale (ωₜ = 0.67).

### Statistical analysis

All analyses were conducted in R (version 4.5.1). Borderline-personality traits were modeled as a continuous moderator: the BPQ total score was standardized (*z*BPQ). The three MentS subscales—Motivation to Mentalize, Other-Related Mentalization, and Self-Related Mentalization—were standardized (*z*-scores) before entry into the models. We adjusted all regression models for age, gender, educational level, socioeconomic status, and psychiatric diagnosis. Missing data were handled by pairwise deletion for correlations and listwise deletion for regressions.

We first described zero-order relations among BPQ, psychopathy (LSRP total, primary and secondary), and MentS variables in the full sample using Pearson correlations. The primary inferential test used linear regression. For each outcome (LSRP primary, LSRP secondary), we fit two nested models: a main-effects model including the three MentS subscales and *z*BPQ together with the covariates, and an interaction model that added the three product terms (Motivation × *z*BPQ, Other-Related × *z*BPQ, Self-Related × *z*BPQ) while retaining the same covariates. Within the interaction model we interpreted unstandardized coefficients (*b*), standard errors (SE), *t* values, and *p* values, and summarized fit with R^2^ and adjusted R^2^.

To aid interpretation of significant interactions, we computed simple slopes of each MentS predictor at *z*BPQ = −1 SD, 0, and +1 SD by combining the MentS main-effect coefficient with the corresponding interaction coefficient evaluated at those moderator levels. For graphical displays of moderation, predictions were generated across the observed range of the focal MentS predictor at *z*BPQ = −1, 0, and +1 SD, while holding non-focal MentS predictors at their sample means and fixing covariates at typical values (standardized covariates at 0; categorical covariates at their sample mode). We visualized regression coefficients and 95% CIs using forest plots and inspected standard regression diagnostics; variance inflation factors were examined. As an optional probe, Johnson–Neyman intervals were calculated to examine the simple slope of Motivation across levels of *z*BPQ, the function should be called with Motivation as the focal predictor, and *z*BPQ as the moderator.

## Results

### Severity and scores

In the full sample (*N* = 953), borderline traits were modest on average (BPQ: *M* = 20.84, SD = 13.56). Psychopathy levels were in the moderate range on average *M* = 52.83 (SD = 10.86) for the LSRP total, with *M* = 29.81 (SD = 8.19) for primary psychopathy and *M* = 23.02 (SD = 4.42) for secondary psychopathy. Mentalization was moderate overall (MentS total: *M* = 94.25, SD = 12.34); by subscale, Motivation to Mentalize averaged *M* = 30.42 (SD = 4.83), Other-Related Mentalization *M* = 35.87 (SD = 5.36), and Self-Related Mentalization *M* = 27.96 (SD = 6.16).

### Correlation coefficients among study variables

As showed in Table 3, borderline traits (BPQ total) correlated positively with psychopathy (BPQ with LSRP total, *r* = 0.60; with primary, *r* = 0.46; with secondary, *r* = 0.63). Mentalization was inversely related to psychopathy (MentS total with LSRP total, *r* = −0.44; with primary, *r* = −0.38; with secondary, *r* = −0.37) and to BPQ (*r* = −0.36). For the MentS subscales, Motivation to Mentalize correlated weakly and negatively with psychopathy (with LSRP total, *r* = −0.29; with primary, *r* = −0.30; with secondary, *r* = −0.17) and with BPQ (*r* = −0.10). Other-Related Mentalization showed small negative correlations with psychopathy (with LSRP total, *r* = −0.26; with primary, *r* = −0.24; with secondary, *r* = −0.19) and with BPQ (*r* = −0.22). Self-Related Mentalization showed moderate negative correlations with psychopathy (with LSRP total, *r* = −0.42; with primary, *r* = −0.32; with secondary, *r* = −0.44) and with BPQ (*r* = −0.44). As expected, MentS total correlated strongly with the two MentS subscales (MentS with Other, *r* = 0.80; MentS with Self, *r* = 0.71), and the MentS subscales correlated with each other (*r* = 0.58 and *r* = 0.24–0.28). All *ps* shown in the correlation table were < 0.001.

**Table 3:** Correlation matrix for study variables.

	1	2	3	4	5	6	7	8
1. BPQ	—							
2. LSRP	0.60^***^	—						
3. Primary psychopathy	0.46^***^	0.93^***^	—					
4. Secondary psychopathy	0.63^***^	0.73^***^	0.43^***^	—				
5. MentS	−0.36^***^	−0.44^***^	−0.38^***^	−0.37^***^	—			
6. Motivation	−0.10^***^	−0.29^***^	−0.30^***^	−0.17^***^	0.76^***^	—		
7. Other-Related	−0.22^***^	−0.26^***^	−0.24^***^	−0.19^***^	0.80^***^	0.58^***^	—	
8. Self-Related	−0.44^***^	−0.42^***^	−0.32^***^	−0.44^***^	0.71^***^	0.24^***^	0.28^***^	—

^***^p < 0.001, BPQ, Borderline Personality Questionnaire; LSRP, Levenson Self-Report Psychopathy Scale; MentS, Mentalization Scale.

### Multivariable predictors of psychopathy

#### Primary psychopathy, adjusted

In the model including BPQ (*z*-scored), the three MentS predictors, and covariates (age, gender, socioeconomic status, psychiatric diagnosis), higher Motivation (*b* = −0.35, SE = 0.06, *t* = −6.34, *p* < 0.001) and higher Self-Related Mentalization (*b* = −0.18, SE = 0.04, *t* = −4.45, *p* < 0.001) uniquely predicted lower primary psychopathy; Other-Related Mentalization was not significant (*b* = 0.04, SE = 0.05, *t* = 0.85, *p* = 0.40). BPQ showed a positive association (*b* = 2.52, SE = 0.25, *t* = 9.88, *p* < 0.001). Among covariates, younger age (*b* = −1.82, SE = 0.23, *t* = −8.05, *p* < 0.001), female gender vs. male (*b* = 3.22, SE = 0.44, *t* = 7.27, *p* < 0.001), and psychiatric diagnosis (*b* = 1.39, SE = 0.59, *t* = 2.34, *p* = 0.02) were significant. Model fit: *R*^2^ = 0.37, adj. *R*^2^ = 0.37, *F*_(9, 943)_ = 62.28, *p* < 0.001.

#### Secondary psychopathy, adjusted

Self-Related Mentalization (*b* = −0.14, SE = 0.02, *t* = −6.71, *p* < 0.001) and Motivation (*b* = −0.09, SE = 0.03, *t* = −3.07, *p* = 0.002) predicted lower secondary psychopathy; Other-Related Mentalization was not significant (*b* = 0.02, SE = 0.02, *t* = 0.96, *p* = 0.34). BPQ was positively associated (*b* = 2.40, SE = 0.13, *t* = 18.46, *p* < 0.001). Higher socioeconomic status was modestly associated with lower scores (*b* = −0.22, SE = 0.11, *t* = −1.98, *p* = 0.048). Model fit: *R*^2^ = 0.44, adj. *R*^2^ = 0.43, *F*_(9, 943)_ = 81.72, *p* < 0.001.

#### Moderation by borderline traits

**Figure 1:**
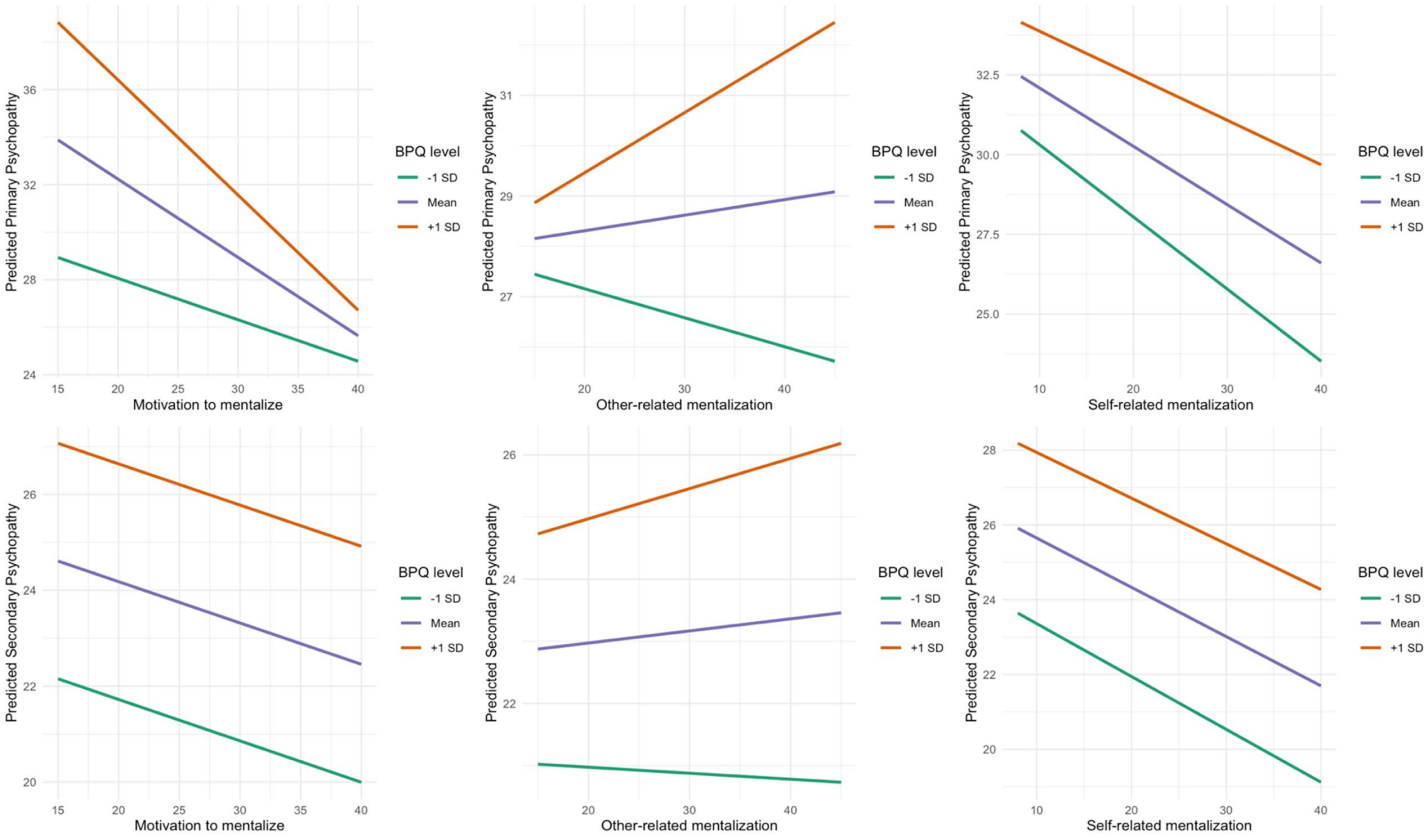
Conditional effects of mentalization on primary and secondary psychopathy across borderline trait Levels. BPQ, Borderline Personality Questionnaire.

Figure 1 shows simple slopes of mentalization facets predicting psychopathy at low (−1 SD), mean, and high (+1 SD) BPQ levels. For primary psychopathy, the Motivation × BPQ interaction was significant: *b* = −0.16, SE = 0.05, *t* = −3.07, *p* = 0.002. Simple-slope estimates for Motivation at BPQ = −1/0/+1 SD were −0.17 / –0.33 / –0.48, indicating a steeper protective slope at higher BPQ. The Other × BPQ term approached but did not reach significance (*b* = 0.09, SE = 0.05, *t* = 1.89, *p* = 0.060); corresponding simple-slope estimates at BPQ = −1/0/+1 SD were 0.00 / 0.02 / 0.05. The Self × BPQ term was not significant (*b* = 0.04, SE = 0.04, *t* = 1.17, *p* = 0.243), with simple slopes of −0.14 / –0.13 / –0.12. Overall model fit for the adjusted interaction model was *R*^2^ = 0.380, adj. *R*^2^ = 0.372, *F*_(12, 940)_ = 47.93, residual SE = 6.49. For secondary psychopathy, none of the interaction terms reached significance: Motivation × BPQ (*b* = 0.00, SE = 0.03 *t* = 0.01, *p* = 0.994), Other × BPQ (*b* = 0.03, SE = 0.02, *t* = 1.21, *p* = 0.227), Self × BPQ (*b* = 0.01, SE = 0.02, *t* = 0.51, *p* = 0.614). The adjusted interaction model fit was *R*^2^ = 0.440, adj. *R*^2^ = 0.433, *F*_(12, 940)_ = 61.53, residual SE = 3.33. Corresponding simple-slope estimates were flat or small in magnitude: Motivation −0.09 / –0.09 / –0.09, Other −0.01 / 0.02 / 0.05, Self −0.14 / –0.13 / –0.12 at BPQ = −1/0/+1 SD (see Table 4).

**Table 4:** Moderated regression of psychopathy on mentalization, borderline traits, and their interactions.

Predictor	Primary psychopathy				Secondary psychopathy			
	b	SE	*t*	*p*	b	SE	*t*	*p*
Intercept	41.48	1.89	21.95	< 0.001	28.80	0.97	29.73	< 0.001
Motivation to Mentalize (*z*)	−0.33	0.06	−5.89	< 0.001	−0.09	0.03	−3.00	0.003
Other-Related Mentalization (*z*)	0.03	0.05	0.62	0.538	0.02	0.03	0.76	0.450
Self-Related Mentalization (*z*)	−0.18	0.04	−4.60	< 0.001	−0.13	0.02	−6.46	< 0.001
BPQ (*z*)	2.89	1.54	1.88	0.061	1.14	0.79	1.45	0.148
Age (*z*)	−1.80	0.22	−7.99	< 0.001	−0.05	0.12	−0.39	0.696
Gender: Female vs. male	3.19	0.44	7.23	< 0.001	−0.43	0.23	−1.89	0.059
Gender: Other vs. male	6.84	6.56	1.04	0.298	−1.59	3.37	−0.47	0.637
Socioeconomic status (*z*)	0.35	0.22	1.61	0.107	−0.18	0.11	−1.65	0.100
Psychiatric diagnosis (1 = yes)	1.35	0.59	2.29	0.022	0.09	0.30	0.29	0.771
Motivation × BPQ	−0.16	0.05	−3.07	0.002	0.00	0.03	0.01	0.994
Other × BPQ	0.09	0.05	1.89	0.060	0.03	0.02	1.21	0.227
Self × BPQ	0.04	0.04	1.17	0.243	0.01	0.02	0.51	0.614

Model fit, *R*² = 0.380, adj. *R*² = 0.372, *F*_(12, 940)_ = 47.93, *p* < 0.001. *R*² = 0.440, adj. *R*² = 0.433, *F*_(12, 940)_ = 61.53, *p* < 0.001.

In summary, higher Motivation to Mentalize shows a stronger negative association with primary psychopathy as borderline traits increase, while no such moderation emerges for secondary psychopathy, which is consistent with the pattern of simple slopes and the non-significant interaction tests.

## Discussion

This study offers a novel contribution to the literature by examining the associations between three core dimensions of mentalizing (i.e., Self-Related, Other-Related, and Motivation to Mentalize) and psychopathy subtypes (primary and secondary), as well as testing the moderating role of borderline personality traits. The findings highlight that mentalizing is not merely a cognitive capacity but a multidimensional process that is shaped by distinct personality configurations. Specific patterns emerged linking psychopathy subtypes to mentalizing components, and borderline traits moderated only the association between Motivation to Mentalize and primary psychopathy; no moderation effects emerged for secondary psychopathy.

Consistent with prior literature, the observed negative association between primary psychopathy and Other-Related Mentalizing aligns with prior findings suggesting diminished responsiveness to affective cues and reduced amygdala activation in such individuals (Blair et al., 2004; Bo et al., 2023; Song et al., 2023; Spytska, 2024). In bivariate analyses, Self-Related and Other-Related Mentalizing showed comparable small–moderate negative correlations with primary psychopathy (Self: *r* = −0.32; Other: *r* = −0.24). In adjusted models, Self-Related Mentalizing uniquely predicted lower primary psychopathy, whereas Other-Related Mentalizing did not contribute uniquely once covariates and the other mentalizing facets were included. However, emerging evidence indicates that individuals high in primary psychopathy may retain intact or even elevated cognitive perspective-taking abilities, often deployed manipulatively (Baskin-Sommers and Brazil, 2022; Lukacs et al., 2022). These findings support theoretical models that view mentalizing not only as a skill but as a process influenced by motivational orientation (Dimitrijević et al., 2018; Fonagy et al., 2018). This aligns with findings that individuals with psychopathic traits may strategically utilize their social cognitive abilities for instrumental rather than prosocial purposes (Nentjes et al., 2015). However, in adjusted models that included all mentalizing facets and covariates, Other-Related Mentalizing did not uniquely predict either psychopathy subtype.

The negative association observed between secondary psychopathy and Self-Related Mentalizing is also consistent with this subtype’s core characteristics, including emotional volatility, impulsivity, and high internal distress (Bateman and Fonagy, 2016; Sharp et al., 2011). These individuals often experience difficulties identifying and regulating their own emotional states, which may contribute to externalizing behaviors such as aggression. Prior studies have shown that limited mentalizing capacity is linked to heightened aggression and reduced behavioral control, whereas improved self-reflective capacity may act as a protective factor (Taubner et al., 2013; Velotti et al., 2021). In the present study, greater Self-Related Mentalizing was associated with lower secondary psychopathy, consistent with the view that strengthening self-reflective capacity may buffer impulsive and emotionally reactive tendencies. These findings suggest that therapeutic strategies aimed at enhancing emotional awareness and internal state understanding may be especially relevant for this group.

Findings related to the Motivation to Mentalize dimension further elucidate the distinct profiles of psychopathy. Specifically, individuals with primary psychopathic traits demonstrated a pattern of “instrumental but non-empathic” mentalizing (De Wit-De Visser et al., 2023; Tillem et al., 2021). They tended to ignore emotional content while selectively attending to cognitive and strategic information. This pattern supports models that conceptualize mentalizing as a motivationally driven process rather than a purely cognitive capacity (Dimitrijević et al., 2018; Fonagy et al., 2018). The distinction between cognitive empathy (inferring others’ thoughts) and emotional empathy (resonating with others’ emotions) is especially pertinent here (Sharp et al., 2011). Indeed, Nentjes et al. (2015) found that individuals high in psychopathy may utilize their mentalizing skills not for interpersonal attunement, but for strategic advantage. These patterns underscore the importance of recognizing mentalizing not as universally prosocial but as subject to instrumental and context-dependent usage. Supporting this, recent evidence indicates that therapists working with antisocial individuals often experience therapeutic pessimism, which may stem from failing to appreciate these motivational dynamics (Flaaten et al., 2024). Our pattern (i.e., stronger Motivation linked to lower psychopathy, with no unique effect of Other-Related Mentalizing) aligns with motivational accounts of social cognition, though our measures do not directly test selective strategic use.

A novel contribution of this study is its systematic examination of how borderline personality traits moderate the relationship between mentalizing dimensions and psychopathy subtypes. Our findings indicate that borderline traits moderated the link between Motivation to Mentalize and primary psychopathy only; no evidence of moderation emerged for secondary psychopathy. At higher levels of borderline traits, the inverse association between Motivation and primary psychopathy was steeper, whereas associations involving Self- and Other-Related Mentalizing were not moderated. Borderline traits significantly moderated the link between Motivation to Mentalize and primary psychopathy (*b* = −0.16, SE = 0.05, *t* = −3.07, *p* = 0.002). Simple slopes indicated a progressively stronger inverse association at higher borderline trait levels (−1 SD: −0.17; mean: −0.33; +1 SD: −0.48). Borderline traits may not only act as risk factors but also serve as phenotypic variants that intensify psychopathic tendencies (Chapman, 2019; Daurio and Taylor, 2022).

### Clinical implications

Based on our results and existing literature, interventions targeting psychopathic individuals should be tailored to their personality profiles. For individuals high in primary psychopathy, particularly when borderline traits are elevated, interventions may benefit from explicitly enhancing motivation to consider mental states alongside affective perspective-taking and emotional awareness training. In contrast, for individuals with secondary psychopathy, which is characterized by poor emotional regulation and internal chaos, therapeutic efforts should focus on strengthening self-reflective capacity and emotion regulation strategies (Bateman and Fonagy, 2016; Taubner et al., 2013). For secondary psychopathy, focusing on self-reflective capacity and emotion regulation appears warranted irrespective of borderline trait level. In cases where borderline features are also present, group-based MBT or emotionally focused therapies may be especially beneficial in promoting the safe expression and regulation of affective experiences (Bateman and Fonagy, 2008; Fonagy and Luyten, 2009). When borderline traits are elevated, emphasizing motivation to consider mental states may be especially impactful for reducing primary psychopathic tendencies.

Additionally, improving mentalizing in antisocial individuals may foster not only behavioral change but also enhance therapists’ optimism and engagement in treatment (Flaaten et al., 2024). This points to the importance of conceptualizing interventions not only at the individual level but also in terms of the therapeutic alliance and clinician attitudes. Consistent with adjusted models, younger age, female gender, and psychiatric diagnosis were associated with higher primary psychopathy, whereas higher socioeconomic status was associated with lower secondary psychopathy.

### Strength, limitations, and future directions

The present study benefits from several notable strengths. First, the medium-large community sample affords adequate statistical power to detect small-to-moderate effects and enhances the generalizability of the findings. Second, all constructs were measured with validated, widely used instruments, which support comparability and replicability. Finally, by stratifying and moderating analyses according to borderline personality trait severity, the study offers nuanced insights into personality–psychopathy dynamics that extend prior work focused on undifferentiated samples. Interpretation of the findings must be considered in light of two primary limitations. First, the cross-sectional design precludes inferences about temporal precedence or causal direction among mentalizing, psychopathy, and borderline traits. Second, reliance on self-report measures, although well validated (Stefana et al., 2025), may be subject to self-presentation bias; the absence of clinician-rated or interview-based assessments precludes multimethod convergence. Future research should longitudinally examine changes in mentalizing dimensions over time and explore how these changes relate to clinical outcomes across different psychopathy profiles, especially when comorbid borderline features are present. Qualitative studies exploring therapists’ expectations, biases, and intervention choices may also enrich our understanding of how to best engage with complex personality configurations in treatment settings.

## Conclusion

Taken together, our findings indicate that stronger mentalizing, especially motivation and self-related facets, is associated with fewer psychopathic traits, and the protective effect of motivation is most pronounced for primary psychopathy at higher levels of borderline personality features. Mentalizing is not a fixed trait but a dynamic, context-sensitive process shaped by motivation and personality.

## Data Availability

Data supporting the findings of this study are available from the corresponding author upon reasonable request.
